# Possible Health Implications and Low Vitamin D Status during Childhood and Adolescence: An Updated Mini Review

**DOI:** 10.1155/2010/472173

**Published:** 2009-08-11

**Authors:** Dimitrios Papandreou, Pavlos Malindretos, Zacharoula Karabouta, Israel Rousso

**Affiliations:** ^1^2nd Department of Pediatrics, Aristotle University of Thessaloniki, School of Medicine, Ahepa General Hospital, St. Kiriakidi 1, 54636 Thessaloniki, Greece; ^2^Department of Pathology, Aristotle University of Thessaloniki, School of Medicine, Ahepa General Hospital, St. Kiriakidi 1, 54636 Thessaloniki, Greece

## Abstract

Vitamin D deficiency is common in the developing countries and exists in both childhood and adult life. The great importance of Vitamin D is the moderation of calcium (Ca) and phosphorus (P) homeostasis as well as the absorption of Ca. While insufficiency of vitamin D is a significant contributing factor to risk of rickets in childhood, it is possible that a more marginal deficiency of vitamin D during life span contribute to osteoporosis as well as potentially to the development and various other chronic diseases such as cardiovascular disease, cancer and diabetes. This paper reviews the metabolism, epidemiology, and treatment of vitamin D and calcium insufficiency as well as its relation to various diseases during childhood and adolescence.

## 1. Introduction

### 1.1. Vitamin D Metabolism

Vitamin D has two different forms: Vitamin D2 which exists in food (plants) and D3 (cholecalciferol) which is produced by following the path 7-dehydrocholesterol → previtamin D → vitamin D3 in the skin upon ultraviolet B (UVB) sun exposure and at skin temperature. The process of generating vitamin D3 from sun exposure is attenuated by reduced exposure of the skin to sunlight (northern latitudes with decreased direct sun exposure, air pollution, confinement indoors, clothes covering all the skin, broad use of sunscreens, increased skin pigmentation) or in dermatologic conditions such as ichthiosis in which sun inadequately penetrates the epidermis [1] . The two vitamin D molecules differ in structure; vitamin D2 has an extra double bond between carbons 22 and 23 and an additional 24-methyl group in comparison with vitamin D3 having been demonstrated to be two to three times more effective than vitamin D2 [2–4]. Both forms of the vitamin D diffuses into the circulation and is transported protein-bound to the liver where it is hydroxylated to 25(OH)D3 (calcitriol) and 25(OH)D2. Serum or plasma 25(OH)D3 is the most commonly used and appropriate biochemical marker of vitamin D status [5, 6]. In the kidney, 25(OH)D3 undergoes a further hydroxylation at the first carbon, catalysed by 1,2-hydroxylase, to form 1,25(OH)2D3 which is the biologically most active form of vitamin D [6]. Although 1,25(OH)2D3 represents the active form of the vitamin, due to a tight regulation of its production as well as a relatively short half-life (4–6 hours), it is not a good indicator of vitamin D status [5–7]. Serum 25(OH)D3 concentrations are the best indicator of determining adequacy because it represents the combined amounts of vitamin D synthesized in the skin and dietary sources [8]. Serum 25(OH)D3 levels are the accepted measure of vitamin D nutritional status [9].

### 1.2. Prevalence of Vitamin D Deficiency

In the USA, cases of nutritional rickets have been reported from at least 17 states, with 166 cases reported in the medical literature between 1986 and 2003 [10]. Relatively high rates of subclinical vitamin D deficiencies have been reported in otherwise healthy infants [11–14] children [15, 16] and adolescents [17, 18] in several American states. A high prevalence of vitamin D deficiency has also been reported in infants, children, and adolescents from diverse countries around the world, including the UK [19], France [20], Greece [21], Lebanon [22], Turkey [23], China [24], Finland [25, 26], Canada [27, 28] and Qatar [29]. In the research that took place in China, 18% of total 42 healthy infants were found with rickets and the fact that not all infants with rickets in this study had low 25(OH)D concentrations suggests 2 possibilities: (1) not all rickets is necessarily related to a vitamin D deficiency, or (2) serum 25(OH)D concentrations are not the best indicator of vitamin D status [30]. Among adolescence, reported prevalence rates of vitamin D deficiency have ranged from 0 to 42%, with variation noted secondary to season, latitude, and participant race/ethnicity [31]. Data from national surveys in the UK, USA and New Zealand show that the prevalence of low vitamin D status is less of a concern for children than for adolescents [32–34]. There is also some suggestion that intakes of vitamin D may be worse in females than males. For example, Moore et al. reported that adolescent males in the USA were the group most likely to consume the adequate intake value for vitamin D, while adolescent females were about half as likely as males of corresponding age to meet their dietary reference intakes [35].

 Vitamin D status changes were also reported using data from the National Health and Nutrition Examination Surveys (NHANES). The authors concluded that the mean serum 25(OH)D was lower in 2000–2004 than 1988–1994 and that the assay changes unrelated to changes in vitamin D status accounted for much of the difference in most population groups. They also reported that in the adult subgroup, combined changes in BMI, milk intake, and sun protection appeared to contribute to a real decline in vitamin D status [36]. In addition, Bener et al. [29] recently found a very high incidence of vitamin D deficiency in the young population in Qatar especially in girls. This deficiency attributed by the authors as a result from a combination of limitation in sunlight exposure and a low oral intake of vitamin D. Also, children with renal failure are at risk for vitamin D deficiency. Recently Ali et al. [37] studied over a period of 10-years the prevalence in children with vitamin D efficiency and he found rates from 20% to 75%.

## 2. Health Implications of Vitamin D Deficiency

Increasing evidence suggests that optimal vitamin D status throughout the lifespan—even in utero—may be important not only in maintaining bone health, but also in protecting against many chronic conditions, including autoimmune diseases, diabetes, cardiovascular diseases, and cancer [38].

### 2.1. Rickets

Rickets was a very common disease during the industrialization and had a prevalence of 40%–60% in children living in northern Europe [39]. Rickets was cured after people were informed about the value of vitamin D in bone conformation and the adequate exposure to sunlight as a prohibitive and therapeutic method. Cod liver oil and fortification of infant formula also helped on this direction. The problem began to reappear in 1960, especially among breastfed infants and in those infants whose mothers' dress included covering [40]. Over the past 20–30-years, there has been a reemergence of rickets with reports in the UK [41–43], Europe [44–46],North America [47] and Saudi Arabia [48] in a variety of ethnic groups. The resurgence of rickets in North America in the 1990s coincided with skin cancer prevention campaigns [49]. Studies in South Africa and Nigeria suggest that a dietary deficiency of calcium may cause rickets [50, 51] and there are case reports of rickets caused by dietary calcium deficiency in North America [52, 53]. Most of the children in these studies had normal serum 25-hydroxyvitamin D concentrations and high serum 1,25-dihydroxyvitamin D concentrations, indicating adequate intake of vitamin D. Other possible reasons for the increasing prevalence of rickets include: breastfeeding without vitamin D supplementation, vegetarian diets [54], darker-skinned people migrating to countries with less sunlight [45, 55, 56] and increasing atmospheric pollution [57]. Typically, rickets associated primarily with vitamin D deficiency presents during the first year of life.

### 2.2. Vitamin D Status and Type 1 Diabetes Mellitus (T1DM)

T1DM is among the most prevalent chronic diseases with onset in childhood, being the second most common chronic disease in children after asthma in the USA. It results from an immune-mediated destruction of pancreatic insulin-producing *β*-cells, with both genetic and environmental factors playing roles in the aetiology. It is linked about 60% to genes in the HLA complex of the major histocompatibility complex (MHC) on chromosome 6p21 [58, 59]. Non-MHC chromosomal regions are also involved, particularly the regulatory region of the insulin gene and the interleukin-1 receptor type 1 gene. The specific factors that initiate the autoimmune process are not yet well understood, but *β*-cell destruction often begins during infancy and continues over many months or years [60]. By the time T1DM is diagnosed, about 80% of the b cells have been destroyed. There is a marked geographic variation in incidence, with a child in Finland being about 400 times more likely than a child in Venezuela to acquire the disease [60]. The pattern follows a latitudinal gradient that is the inverse of the global distribution of ultraviolet B (UVB) irradiance. It is estimated that currently the incidence is increasing by 3% per year. Furthermore, it is predicted that by 2010 the incidence of T1DM will be 40% higher than it was a decade earlier [60].

 One of the environmental factors thought to be protective against the development of T1DM, is early supplementation with vitamin D. Vitamin D is either produced endogenously, through skin exposure to sunlight, or exogenously from ingestion of foods and supplements [59, 60]. T1DM incidence rates are higher in regions that are more distant from the equator, where UVB irradiance is lower, than in those closer to the equator, where UVB irradiance is much higher. Although the importance of vitamin D for preventing rickets and adult bone disease is well established, it is becoming increasingly clear that vitamin D appears to be an immunosuppressive agent, a role that may explain its protective association with autoimmune conditions, including multiple sclerosis and rheumatoid arthritis. Strong evidence of a vitamin D effect on T1DM risk comes from experiments in the nonobese diabetic (NOD) mouse. The NOD mouse experiences disease pathogenesis similar to the human, including autoimmune destruction of *β*-cells. When 1,25-dihydroxyvitamin D(1,25(OH)2D), the active form of the vitamin, was administered to NOD mice in pharmacologic doses, it prevented the development of diabetes. More recently, NOD mice raised in a vitamin D deficient state were shown to develop diabetes at an earlier age than nondeficient NOD controls [59–61]. The dependence of normal insulin secretion in pancreatic *β*-cells on vitamin D has been known for several decades. A reduction in vitamin D activity can result in both increased insulin resistance and reduced insulin secretion. Epidemiological data have shown a four-to five-fold higher prevalence of noninsulin-dependent diabetes in dark-skinned Asian immigrants in comparison with British Caucasians indicating that low vitamin D status may contribute to the pathogenesis of diabetes [62].

 A recent review including studies from many European countries, particularly the EURODIAB [63], and the Finnish study [64] in 2001, concluded that there is evidence from observational studies that vitamin D supplementation in infancy might be protective against the development of T1DM. The EURODIAB study found that children whose mothers consumed vitamin D supplements during pregnancy had a lower risk of type 1 diabetes than those whose mothers did not, and indicated that children being supplemented had a 29% reduction in risk of developing type 1 diabetes compared with their peers who were not being supplemented. The favourable association with vitamin D persisted after adjustment for birthweight, duration of breast feeding, maternal age and study centre [61]. A Norwegian study done by Stene and jones [65] in 2003 looked at the effect of the time of starting supplementation with vitamin D. It appears that those who had cod liver oil, an important source of both vitamin D and the long-chain n-3 fatty acids docosahexaenoic acid (DHA)and eicosapentaenoic acid (EPA) in the Norwegian population between 7 and 12 months of age had lower chances of developing T1DM in later life compared to those who were supplemented between 0 and 6 months of age. More recently, significant vitamin D deficiency was found in more than 75% of 128 children with type 1 diabetes [66]. Epidemiological studies describing a north south gradient in incidence rate and an inverse correlation between incidence and mean monthly sunshine hours are also hinting at a possible protective effect of vitamin D [62, 63]. In conclusion, the evidence shows that the vitamin D system plays an important role in T1DM. By interfering with environmental factors, such as sun exposure and subsequent vitamin D levels, there may be an opportunity to prevent some of the cases of T1DM. So, the provision of adequate levels of vitamin D is an important goal for public health [61]. Considering the rapid increase in type 1 diabetes incidence among 0–5-year olds and early appearance of IA, maternal intake of certain dietary nutrients during pregnancy including vitamin D through food, may provide sufficient in utero exposure to these nutrients, offering early protection from or promotion of islet autoimmunity (IA) in infancy or early childhood [63, 67, 68].

### 2.3. Vitamin D Status and Other Diseases

While rickets and osteomalacia are the index diseases for severe vitamin D deficiency, there has been growing evidence that less severe deficiency, may also contribute to other chronic diseases, such as cardiovascular disease, hypertension, cancer and other chronic disease.

 The active form of vitamin D, 1,25-dihydroxyvitamin D, is made in many different tissues, including colon, prostate, and breast. It is believed that the local production of 1,25(OH)2D may be responsible for the anticancer benefit of vitamin D. In a recent review by Holick [69] suggested that women who are vitamin D deficient have a 253% increased risk for developing colorectal cancer, and women who ingested 1500 mg/d calcium and 1100 IU/d vitamin D(3) for 4-years reduced risk for developing cancer by >60%.

 Low 25-hydroxyvitamin D levels have also been associated with the cardiovascular disease risk factors of hypertension, obesity, and the metabolic syndrome, as well as cardiovascular disease events including stroke and congestive heart failure [70]. Studies suggest vitamin D deficiency may be a contributor to the development of cardiovascular disease potentially through associations with diabetes or hypertension [70]. However, much of the evidence base for this comes from epidemiologic studies of adults. These have been expertly reviewed by Holick [71] and Zittermann [62]. Far less is known of the effect of poor vitamin D status during childhood and adolescence on risk of these nonskeletal chronic diseases. However, in his recent review of childhood vitamin D deficiency, Holick highlights evidence that living at latitudes above 35° for the first 10-years of life increases risk of multiple sclerosis by 100%, as well as increasing the risk of several other autoimmune diseases [72]. There is also some evidence that supplementation with vitamin D (usually high dose (20–2500 mg/day) and in some cases in combination with calcium; (low dose vitamin D supplementation did not appear to be effective) may beneficially influence muscle function, rheumatoid arthritis, blood pressure, blood glucose and insulin levels [62].

 After nearly 20-years of debate, there is now sufficient, reproducible evidence supporting a beneficial role for calcium and vitamin D-rich dairy foods in blood pressure regulation [73]. The incidence of hypertension also followed an inverse relation with serum 25(OH)D [74, 75] and increased intake of vitamin D was associated with 9.3 percent decrease in systolic blood pressure in adults [76], while a more recent study by Saintonge et al. [77] revealed that overweight and obesity in adolescence increases the risk for vitamin D deficiency compared with the normal ones. Recently, a new study by Kremer et al. [78] confirmed that, by examined the relationship of 25 (OH)D status and body fat levels in 90 post pubertal females and concluded that vitamin D insufficiency was associated with increased BF.

## 3. Treatment

Rickets can be treated effectively with vitamin D supplementation. In relation to addressing subclinical vitamin D deficiency and insufficiency, sun exposure and dietary vitamin D intake (including vitamin D fortified foods and supplemental vitamin D use) undoubtedly have important roles. However, the relative importance of these two routes of exposure differs from summer to winter for most people. If sun exposure is sufficient, very little if any vitamin D is required from the diet during summer [79]. It is worth remembering, however, that the production of vitamin D in the skin during summer varies with the geographical location, atmospheric conditions, time spent outdoors, clothing, and skin pigmentation [79] as well as sunscreen use. According to Holick, [80] approximately 30 minutes of skin exposure (without sunscreen) of the arms and face to sunlight can provide all the daily vitamin D needs of the body. When sunlight exposure is limited, dietary intakes of vitamin D, if sufficient, can make a significant contribution to vitamin D status. In particular, at latitudes above 37°N, production of vitamin D3 in winter is virtually zero, because the zenith angle of the sunlight increases in the autumn and winter and consequently, the amount of solar ultraviolet radiation that reaches the Earth's surface is substantially reduced. Therefore, there is an increased reliance on dietary vitamin D for maintaining adequate vitamin D status during winter, and even in summer for those who avidly avoid sunshine exposure. While the US authorities recommend 5 mg vitamin D/day for children and adolescents (aged 1–18 years), [5] respectively, in the UK children aged 1–3 years are recommended 7 mg vitamin D/day while there is no dietary recommendation for vitamin D for subjects aged 4–64-years [72]. This lack of dietary recommendation is on the basis that it is assumed that skin synthesis of vitamin D will generally ensure adequacy which depends on regular exposure to summer sunlight [72]. If individuals have restricted sunlight exposure, then 10 mg/day is recommended. However, vitamin D is rather sparsely represented in the diet, which might explain the low intakes in children and adolescents during winter, as mentioned earlier. Oily fish such as salmon, mackerel and sardines contain high amounts of vitamin D. Cod liver oil is also an excellent source of vitamin D. Some meats may contain 25(OH)D3. Fortified foods can also be a major contributor to dietary vitamin D2 Vitamin D-fortified foods include some types of margarines, breakfast cereals, infant formulae, fruit juices, chocolates and milks, to name but a few. Use of vitamin D-containing supplements can also make a major contribution to mean daily intake of vitamin D in both adults and children.

 While rickets can be prevented with far smaller doses, a level needed to prevent diabetes would require intake, by children aged ≥1 year, of approximately 25–50 *μ*g (1000–2000 IU)/day of vitamin D3, an intake associated with major reduction in incidence in Norway. Such intake has no known adverse health effects in adults. Children aged ≥1 year and who are outdoors in sunlight for a few minutes each day may achieve similar serum levels with smaller oral intake. Pending further research, oral vitamin D intake of infants <1-year old should not exceed 6.25 *μ*g (250 IU)/day. Physicians and nutritionists should advise parents that children ≥1 year who live more than approximately 30° from the equator should consume 25–50 *μ*g (1,000–2,000 IU)/day of vitamin D3, especially during winter, to substantially reduce their risk of childhood type 1 diabetes. It is also very important to point out that supplementation with Vit D3 it is more efficient than Vit D2 for increasing 25(OH)D levels. In a study by Trang et al. [81], 72 subjects took 260 nmol (approximately 4000 IU) vitamin D2 (*n* = 17) or vitamin D3 (*n* = 55) daily for 14 d. The increase in 25(OH)D with vitamin D3 was 23.3 ± 15.7 nmol/L, or 1.7 times the increase obtained with vitamin D2 (13.7 ± 11.4 nmol/L; *P* = .03). More recently, Leventis and Kiely [82], reported that vitamin D3 had greater potency than equimolar vitamin D2, with a higher, sustained serum 25(OH)D response and efficacious PTH suppression.

## 4. Conclusions

There is enough evidence that severe deficiency of vitamin D may lead to skeletal and nonskeletal disease. Both children and adolescents seem to be in high risk of low vitamin D status especially during winter. Having a diet higher in calcium and vitamin D as well as oral supplementation with vitamin D may be necessary for children and adolescents not only in the absence of sun exposure in winter time but also in preventing other diseases such as diabetes type 1, cancer and cardiovascular disease. Further research is needed in order to identify the optimal dietary recommendation needed begging from pregnancy in order to prevent vitamin D deficiency.
